# Skylign: a tool for creating informative, interactive logos representing sequence alignments and profile hidden Markov models

**DOI:** 10.1186/1471-2105-15-7

**Published:** 2014-01-13

**Authors:** Travis J Wheeler, Jody Clements, Robert D Finn

**Affiliations:** 1 HHMI Janelia Farm Research Campus, Ashburn, VA 20147, USA

**Keywords:** Alignment logo, Sequence logo, Profile logo, Hmm logo, Logo server, Web logo

## Abstract

**Background:**

Logos are commonly used in molecular biology to provide a compact graphical representation of the conservation pattern of a set of sequences. They render the information contained in sequence alignments or profile hidden Markov models by drawing a stack of letters for each position, where the height of the stack corresponds to the conservation at that position, and the height of each letter within a stack depends on the frequency of that letter at that position.

**Results:**

We present a new tool and web server, called Skylign, which provides a unified framework for creating logos for both sequence alignments and profile hidden Markov models. In addition to static image files, Skylign creates a novel interactive logo plot for inclusion in web pages. These interactive logos enable scrolling, zooming, and inspection of underlying values. Skylign can avoid sampling bias in sequence alignments by down-weighting redundant sequences and by combining observed counts with informed priors. It also simplifies the representation of gap parameters, and can optionally scale letter heights based on alternate calculations of the conservation of a position.

**Conclusion:**

Skylign is available as a website, a scriptable web service with a RESTful interface, and as a software package for download. Skylign’s interactive logos are easily incorporated into a web page with just a few lines of HTML markup. Skylign may be found at http://skylign.org.

## Background

### Alignments and profile hidden Markov models

Alignments of multiple biological sequences play an important role in a wide range of bioinformatics applications, and are used to represent sequence families that range in size from DNA binding site motifs to full length proteins, ribosomal RNAs, and autonomous transposable elements. In an alignment, sequences are organized such that each column contains amino acids (or nucleotides) related by descent or shared functional constraint. The distributions of letters will typically vary from column to column. These patterns can reveal important characteristics of the sequence family, for example highlighting sites vital to conformation or ligand binding.

A sequence alignment can be used to produce a profile hidden Markov model (profile HMM). Profile HMMs provide a formal probabilistic framework for sequence comparison [1-3], leveraging the information contained in a sequence alignment to improve detection of distantly related sequences [4,5]. They are, for example, used in the annotation of both protein domains [6-9] and genomic sequence derived from ancient transposable element expansions [10].

Consider a family of related sequences, and an alignment of a subset of those sequences. For each column, we can think of the observed letters as having been sampled from the *distribution*, $\vec{p}$ of letters at that position among all members of the sequence family. One approach to estimating $\vec{p}$ for a column is to compute a maximum likelihood estimate directly from observed counts at that column. An alternative is to try to improve the estimate using sequence weighting (relative [11] and absolute [12]) and mixture Dirichlet priors [2,13-15]. The later approach is used in computing position-specific letter distributions for profile HMMs [16,17].

In an alignment, a subset of the columns will be *consensus columns*, in which most sequences are represented by a letter, rather than a gap character. In a typical profile HMM, a model position is created for each consensus column, and non-consensus columns are treated as insertions relative to model positions. As with letters, the per-position gap distributions may be estimated from observed or weighted counts, or combined with a Dirichlet prior.

### Logos

A logo provides a compact graphical representation of an alignment, representing each column with a stack of letters. The total height of each stack corresponds to a measure of the invariance of the column – typically, it is the *information content* of that position. The height of each letter within a stack depends on the frequency of that letter at that position. Logos were originally devised to represent the extent of letter conservation in each column of an alignment [18,19], and were later generalized to show letter and gap probabilities of a profile HMM [20].

Consider an alphabet *A* consisting of *L* letters, *a*_1_ through *a*_*L*_ (*L* is 4 for DNA, and 20 for amino acids). For a given column in an alignment, we capture the estimated column distribution as a length-*L* vector $\vec{p}$, such that *p*_*i*_ is the probability of observing letter *a*_*i*_ at that column. We define the length-*L* vector $\vec{q}$ to be the background distribution over letters in *A*, such that *q*_*i*_ is the background probability of observing letter *a*_*i*_, typically based on letter frequency in a large set of representative sequences.

Given $\vec{p}$ and $\vec{q}$, the *information content*[18] of the column, also called r*elative entropy* or *Kullback–Leibler distance*[15,21], is defined as:

(1) $$D\left(\vec{p}\;|\;\vec{q}\right)=\sum_{1}^{L}\;p_{i}\;\log\left(p_{i}/q_{i}\right).$$

When the base of the log is 2, the information content is expressed in *bits*. This value indicates the extent to which a column’s distribution $\vec{p}$ differs from the background $\vec{q}$, and serves as a measure of the conservation of the column. Information content is non-negative, largest when a column is invariant, and especially large when the invariant letter is rare in $\vec{q}$. For example, the maximum information content for one column in a DNA alignment under uniform background distribution is 2 bits. The maximum for an amino acid alignment under the background corresponding to the BLOSUM62 scoring matrix is roughly 6.5 bits – this for an invariant column of Tryptophan, which has the lowest background probability. Table 1 shows examples of information content values for a few DNA letter distributions, to give some insight into the complex relationship between information content and letter frequencies.

**Table 1:** Relationship between DNA letter distribution and information content

**A%**	**C%**	**G%**	**T%**	**Information content**
100	0	0	0	2.00
95	5	0	0	1.71
90	10	0	0	1.53
85	5	5	5	1.15
80	10	5	5	0.98
70	10	10	10	0.64
50	50	0	0	1.00
50	40	5	5	0.54
45	45	5	5	0.53
50	30	10	10	0.31
35	35	15	15	0.12
25	25	25	25	0.00

Values assume a 4-letter DNA alphabet with a uniform background distribution. The maximum information content under these conditions is 2.0, for an invariant distribution. The minimum value is 0.0, achieved when the letter distribution matches the background. Note that small perturbations away from invariance result in large reductions in information content.

**Figure 1:**
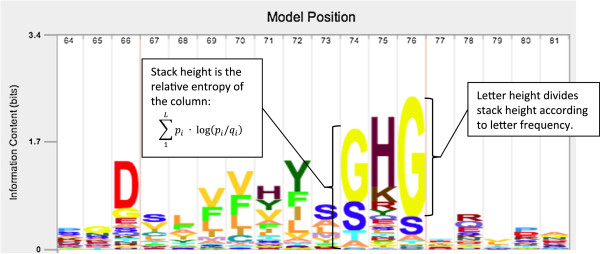
**Example profile logo.** This logo shows positions 64 to 81 of the Peptidase_C14 profile HMM from Pfam (PF00656, Pfam 27.0), produced using Skylign. The profile HMM was constructed using *hmmbuild* (default parameters) from HMMER 3.1 on the Pfam seed alignment. One of the active sites of this Caspase domain is found at position 75. This site is invariant in active peptidases, but not in this profile HMM. This is the result of two forces: (1) the Pfam alignment includes non-peptidase homologs, which do not contain a Histidine at this position, and (2) HMMER intentionally drives down the information content per position (using an approach called entropy weighting [12]) to increase sensitivity to remote homologs.

For a conventional logo, a stack’s height is spread among the letters in alphabet *A* based on $\vec{p}$, such that the height of each letter *a*_*i*_ within a stack is $\left(p_{i}\cdot D\left(\vec{p}|\vec{q}\right)\right)$. Letters are sorted such that those with larger *p*_*i*_ appear near the top in the stack. An example is shown in Figure 1.

## Implementation

We present a software tool and associated web service, called Skylign, which offers several advantages over existing logo tools. It can generate both a static image file and a new interactive web plot that supports scrolling, zooming, and inspection of values underlying each letter stack. Skylign also produces a simplified representation of per-position gap probabilities, and optionally reduces visual clutter by including only overrepresented letters in a stack. Skylign’s interactive logos are robust and fast for alignments with length in the thousands, such as those representing many transposable element families.

An important implementation detail is that Skylign produces logos for both profile HMMs and multiple sequence alignments in a unified framework. Profile logos are plotted using the per-position distributions of the profile HMM. For alignment logos, the column distributions can be estimated either from observed counts, weighted counts, or based on posterior probabilities after combining with a Dirichlet mixture prior. Estimation based on weights and priors is performed by explicitly producing a profile HMM using the *hmmbuild* tool within HMMER3.1 [17].

In the following sections, we describe implementation details, compare alternative visualization approaches, and illustrate the utility of these logos. Skylign can be accessed as a web service at http://skylign.org, and the Skylign software package may be downloaded for independent installation.

## Results and discussion

Several logo web servers have been released since the introduction of logos [20,22-24], each with their own enhancements to logo presentation. In the course of developing websites for sequence homology search and annotation, we identified a need for interactive web-enabled logos that could efficiently render very long logos, and offer alternate letter height options, improved visualization of per-position gap parameters, and the ability to inspect underlying values. We developed Skylign to meet these needs.

### Web-enabled interactive logos

**Figure 2:**
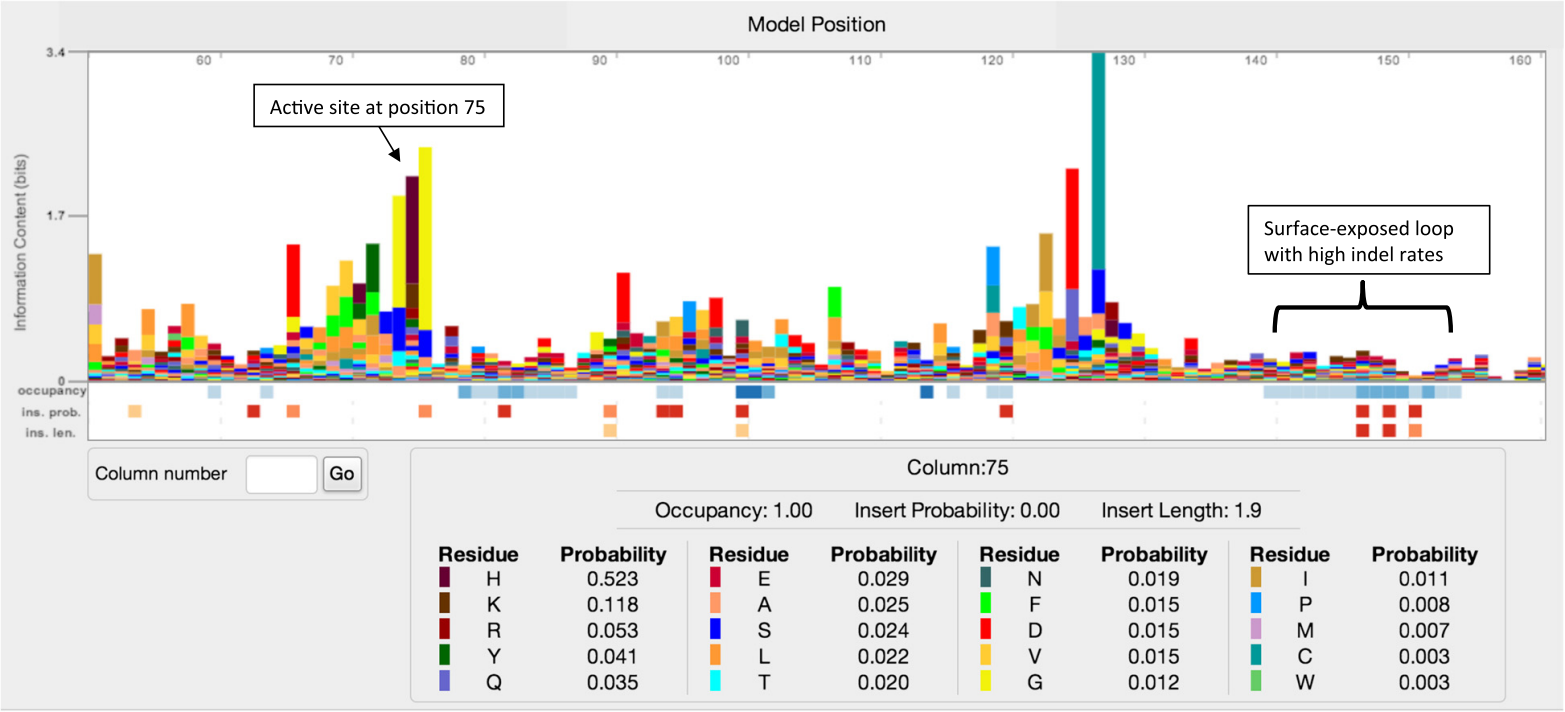
**Compressed overview.** The overview compresses letters into thin vertical bars to enable visualization over a wide section of a logo. Here, we show positions 51 to 160 of the HMM shown in Figure 1. The color of each bar matches the color of the letter represented by that bar. The active sites of this Caspase domain are found at positions 75 (Histidine, shown as the tall brown bar surrounded by yellow bars to the left) and 127 (Cysteine, shown as the taller turquoise bar to the right). The positions around 150 are located in a surface-exposed loop, so it is not surprising to see that they have low sequence conservation and occupancy, and that some have a high insert probability and expected insert length. Below the logo is a table of residue and gap probabilities for position 75, shown as a result of clicking on the corresponding stack – it shows that Histidine (H) is most common, followed by Lysine (K) and Arginine (R).

Historically, all logo software has produced static images (e.g. png or vector graphics files). These are the appropriate formats for inclusion in manuscripts and slides, and may be produced with Skylign, but are suboptimal for distribution on the web. For website integration, Skylign implements interactive logos that support navigation to a requested position in the logo, scroll smoothly, and can be zoomed out for a compressed overview of several hundred positions of the logo. Because profile HMMs create positions only for consensus columns, and because a logo stack is defined only for non-empty alignment columns, not all columns in an alignment will be represented by a position in a profile logo; Skylign optionally shows the mapping between each logo position and the corresponding column in the underlying alignment. Skylign logos also support clicking on individual letter stacks to view the underlying values for all letters, as seen in Figure 2.

The data used to produce an interactive logo is stored as a JSON object, which is rendered using HTML5 Canvas and a custom JavaScript module. Adding a Skylign interactive logo to a web page is simple, requiring the addition of a few lines of markup to the page and reference to the Skylign javascript and css files.

Skylign may be used in a variety of ways to create an image or interactive logo. The simplest option is to use the website submission form. Skylign also offers a web service via a RESTful interface [25], enabling scripted logo creation. Finally, the Skylign package may be downloaded for local installation. Instructions for all of these options are available at http://skylign.org.

### Position-specific gap parameters

In addition to representing the letter distribution at each position, Skylign renders position-specific gap parameters. It does this by presenting up to three values for each position *k*:

1. Occupancy: the probability of observing a letter at position *k*. If we call this value, *occ*(*k*) the probability of observing a gap character (part of a deletion relative to the model) is 1 − *occ*(*k*).

2. Insert probability: the probability of observing one or more letters inserted between the letter corresponding to position *k* and the letter corresponding to position (*k* + 1).

3. Insert length: the expected length of an insertion following position *k*, if one is observed. For mathematical convenience, profile HMMs model insertions as having a geometric length distribution with position-specific parameter *ϵ* and mean length 1/(1 − *ϵ*).

The later two are only relevant for profile logos, since Skylign creates a logo position for each non-empty column in the alignment when producing an alignment logo.

**Figure 3:**
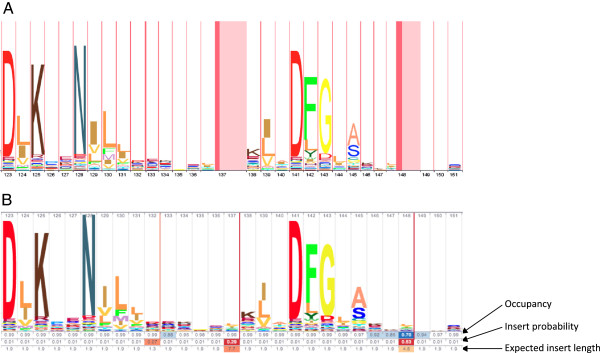
**Example of profile logos from LogoMat and Skylign.** Particular focus is placed on gap parameter visualization, using a section of the Pfam Pkinase protein domain (Pfam accession PF00069). Positions 137 and 148 both have high probability of being followed by an insertion (29% and 53%, respectively), with modest expected insert length (7.7 and 4.5, respectively). Position 148 also has a low occupancy (70%). **(A)** The logo produced by LogoMat, which represents gap parameters by stretching in the horizontal plane. Positions with low occupancy are given less horizontal space, but this is difficult to see. Insert rate and expected length are represented with variable-width red and pink columns. **(B)** Skylign simplifies visual interpretation of gap parameters by presenting a three row table beneath the letter stacks. The top row shows occupancy, with stronger blue background indicating lower occupancy. The middle row presents insert probability, and the bottom row shows expected insert length. For both insert rows, a stronger red background indicates higher values. Note that the default expected insert length (1.9) depends on the priors used when constructing the HMM; observed shorter or longer inserts can shift the expected length away from this value. When a cell in the middle row (insert probability) is not white, a thin red vertical bar of matching color is drawn immediately after the position for that cell, indicating that the insertion will produce letters between the neighboring positions.

The tool LogoMat [20] generalized alignment logos to present these gap parameters for profile HMMs. In LogoMat, occupancy is represented by varying the width of the letter stacks (the stack is thinner for positions with lower occupancy). The insertion probability and expected length are represented by placing variable-width two-toned columns between each letter stack, where the width of the darker part of the column corresponds to the insert rate and the width of the lighter part conflates expected length with insert rate. The result is that gap information is encoded by stretching the horizontal plane. As seen in Figure 3A, column width differences are difficult to discern. In Skylign, stack spacing is uniform, and these parameters are instead represented by up to three rows of numerical values placed below the letter stacks of the logo, with a heat map laid over the top of each value to provide a visual aid. See Figure 3B for an example. This approach – pulling gap information into a distinct section below the letter stacks – bears some similarity to the approach used in the SUPERFAMILY database [6], and simplifies visualization of gap parameters.

### Unified framework for profile logos and alignment logos

**Figure 4:**
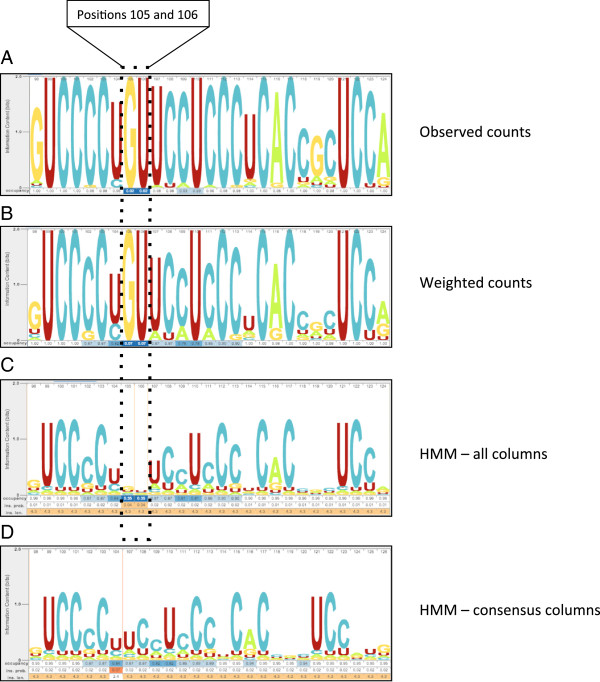
**Comparison of alternate methods of producing an alignment logo from an input sequence alignment.** Logos were built using the Rfam family seed alignment of 45 7SK sequences (RF00100, Rfam 11.0 [26]). Skylign performs necessary counting, weighting, and mixing by explicitly building a profile HMM using the HMMER tool *hmmbuild*. **(A)** Logo for the alignment based on observed frequencies (‘*hmmbuild ‒‒symfrac 0 ‒‒wnone ‒‒enone ‒‒pnone*’). Positions 105 and 106 highlight the fact that Skylign creates a logo position for every non-empty column in the alignment. Occupancy is ~2% because only one of 45 sequences contains a letter at each of those positions. Stacks are tall because stack height depends on observed counts, and there is no variability at these positions. **(B)** Logo for the alignment after applying sequence weighting [11] to account for sequence redundancy (‘*hmmbuild ‒‒symfrac 0 ‒‒pnone*’). The letter G at the first visible position of the weighted logo indicates much less conservation than does the G for the logo based on observed counts. This is because most of the support for high conservation of G at that position comes from a large set of highly similar sequences, and the importance of such redundant sequences is diminished under sequence weighting. Sequence weighting can also alter the represented occupancy rates, for example showing a weighted 7% occupancy for positions 105 and 106. **(C)** Logo for the alignment after applying sequence weighting, absolute weighting [12], and Dirichlet priors. This amounts to building a profile HMM under default HMMER conditions, except that a match state is created for every non-empty column in the alignment (‘*hmmbuild ‒‒symfrac 0’*). In the case of low weighted counts, as in positions 105 and 106, HMMER’s priors typically increase letter variance, leading to lower information content. **(D)** Logo for the HMM built using default ‘*hmmbuild*’, in which logo positions are created only for consensus, resulting in removal of positions 105 and 106.

Skylign can produce a profile logo based on a profile HMM, or an alignment logo based on a sequence alignment, both sharing the same interface. Generating a profile logo is a straightforward matter: a profile HMM stores estimated letter and gap parameters based on the underlying sequence alignment. Skylign simply extracts these values for use in computing stack heights, letter heights, and gap-related values. Alignment logos are more flexible, since Skylign offers four methods for computing estimated distributions from observed frequencies, demonstrated in Figure 4. For all methods, Skylign uses the *hmmbuild* tool from HMMER 3.1 to compute letter and gap values, with alternate option flags used for each method.

### Logo height options

**Figure 5:**
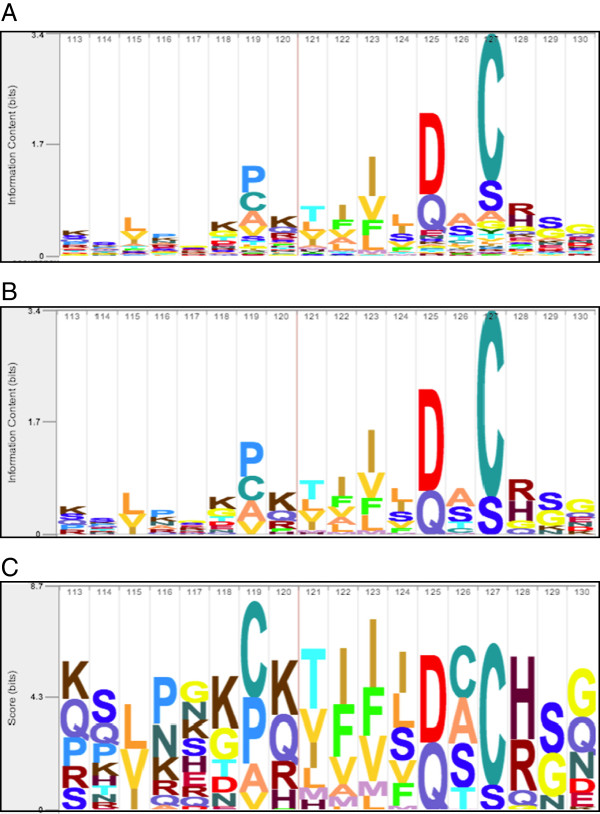
**Comparison of alternate methods of computing letter height within a stack.** These examples were built using the profile HMM for Peptidase_C14 Pfam protein domain family (PF00656), built using the *hmmbuild* tool from HMMER 3.1. **(A)***Information content (all)*: letter stack height is the information content of the column, and all letters subdivide that stack height according to their probability. For models built using strong priors, as with HMMER 3.1, it is common to see an unreadable clutter of below-background letters at the bottom of the stack. See an example under the prominent D and Q at position 125. **(B)***Information content (above-background)*: a less noisy variant, in which stack height is also based on information content, and that height is divided only among letters with above-background probability. Notice the reduced letter clutter in position 125. **(C)***Score*: a variant in which a letter’s height depends on the score of that letter at that position. Only positive-scoring letters (those with above-background probability) are included in the stack. In this case, the height of a stack does not have any inherent meaning – it is simply the sum of all letter heights. As an example of this, note that the stack for position 126 is slightly taller than the stack for position 125, despite the fact that position 125 is much more conserved as seen in Figure 5**A** and **B**. This is because the more conserved 125 has only two positive scoring letters (D=3.4, Q=2.6), while position 126 has five (C=2.0, A=1.8, S=1.7, T=0.8, M=0.04). The stacking order in Figure 5**C** may differ from the order in Figures **A** and **B**. This is because relative letter height in A and B depends only on the frequency in the distribution $\vec{p}$, whereas letter height in 5**C** depends on the score, which accounts for the background distribution, *s*_*i*_ := log _2_(*p*_*i*_/*q*_*i*_).

In the case of protein sequences, when observed counts are combined with a strong Dirichlet mixture prior, the posterior letter distribution often contains small but non-negligible probabilities for all 20 letters. This results in an illegible smear of letters at the bottom of the letter stack of the typical logo (Figure 5A). To address this, Skylign offers an alternate method of computing letter heights for a position, in which the only letters shown in each stack are those with above-background probability. Given the column letter distribution $\vec{p}$ and the background distribution $\vec{q}$, the score of letter *a*_*i*_ in that column is its log odds ratio, *s*_*i*_ := log _2_(*p*_*i*_/*q*_*i*_). Letters with above-background probability will have a positive score. Total stack height is computed in the typical fashion (information content), and the height of the stack is subdivided according to the relative probabilities of the positive-scoring letters, as shown in Figure 5B. In the interactive web logo, clicking a column reveals a list of the probabilities of observing each letter at that position (both above- and below-background letters).

Skylign also offers an option to produce a different sort of logo in which the height of each letter is its score, *s*_*i*_. Only positive-scoring letters are included in the stack, as demonstrated in Figure 5C. We find this logo useful, for example, when inspecting per-position scores of an alignment of a sequence to a profile HMM. It is important to emphasize that the height of a score stack does not have any inherent meaning – it is simply the sum of all letter heights. In the interactive web logo, clicking a column reveals a list of scores for all letters of the alphabet, including those with negative scores.

## Conclusion

Logos have long been used to visually represent the position-specific patterns of conservation in sequence alignments and profile HMMs. We developed Skylign with the aim of enabling interactive manipulation and inspection of logos, while offering a variety of logo variants for alignments and profiles. The result is a logo tool that supports scrolling, zooming, inspection of underlying values, and mapping between logo positions and alignment columns. Skylign simplifies the representation of gap parameters, offers alternate calculations to determine letter heights, and can overcome sampling bias by down-weighting redundant sequences and by combining observed counts with informed priors.

Skylign’s interactive logos are easily incorporated into a web page, and we have already used them in our HMMER and Dfam webservers, presenting logos for both protein and DNA profile HMMs [10,27]. We anticipate that Skylign will be used to create logos, either in advance or on the fly, for other sites that present data related to multiple sequence alignments or profile HMMs.

## Availability and requirements

Skylign can be accessed as a web server and web service, and may be downloaded for local use at http://skylign.org.

## Competing interests

The authors declare that they have no competing interests.

## Authors’ contributions

TJW and RDF motivated creation of interactive web logos with improved gap representation. JC developed the code for logo rendering and the web service. TJW implemented routines in HMMER to produce logo parameters and drafted the manuscript. All authors developed code interfacing the web front end with HMMER back end. All authors read and approved the final manuscript.
